# Supplementary material for: Locally commensurate charge-density wave with three-unit-cell periodicity in YBa2Cu3Oy
Source: Nat Commun. 2021 Jun 1;12:3274. doi: 10.1038/s41467-021-23140-w (PMC8169916; doi:10.1038/s41467-021-23140-w)
Supplement: Supplementary file 1 — Supplementary Information [file 41467_2021_23140_MOESM1_ESM.pdf]

SUPPLEMENTARY INFORMATION FOR

Locally commensurate charge-density wave with three-unit-cell periodicity in $YBa_2Cu_3O_y$

I. Vinograd *et al.*

doping p [holes]	T_c [K]	oxygen content y	chain ordering
0.088	52.3 ± 2	6.49	O-II
0.104	59.6 ± 2	6.54	O-II
0.109	60.0 ± 1	6.56	O-II
0.125	68.5 ± 1	6.68	O-VIII
0.136	78.3 ± 1	6.77	O-III

Supplementary Table 1: Properties of the single crystals studied in this work. The oxygen content of the O-II ordered sample with $p = 0.088$ was determined by ^{63}Cu-NMR in ref. [1].

Supplementary Note 1. Interpretation of earlier Cu NMR results

We further elaborate on the interpretation of initial ^{63}Cu NMR experiments in YBCO in terms of unidirectional CDW, running along the crystallographic a axis, with a commensurate $4a$ period [2]. As explained in the main text, this proposal was aimed at providing a single explanation for two separate observations: a splitting of NMR lines for those Cu sites below oxygen-full chains and the invisibility of this effect for those sites below empty chains in the ortho-II (O-II) structure. The unequal intensity of the split NMR peaks, which is inconsistent with a period 4 (as correctly pointed out in ref. [3]) was attributed to the (observed) small differences in the relaxation time T_2. However, it was not possible to establish how much of this intensity difference stemmed from a T_2 effect as the peaks strongly overlap and T_2 of ^{63}Cu nuclei is very short ($\sim 10\mu$s) in high fields.

There are two conditions for the ^{63}Cu NMR spectra to be consistent with a wave vector $q \simeq 1/3$: 1) the contrasting response below empty and full chains (*i.e.* along a) must have a separate explanation, 2) most of the intensity difference between the split peaks must be an intrinsic property, not a T_2 artefact. The present work uses ^{17}O NMR to show that the unequal intensities of the two peaks indeed result mostly from the period-3 CDW.

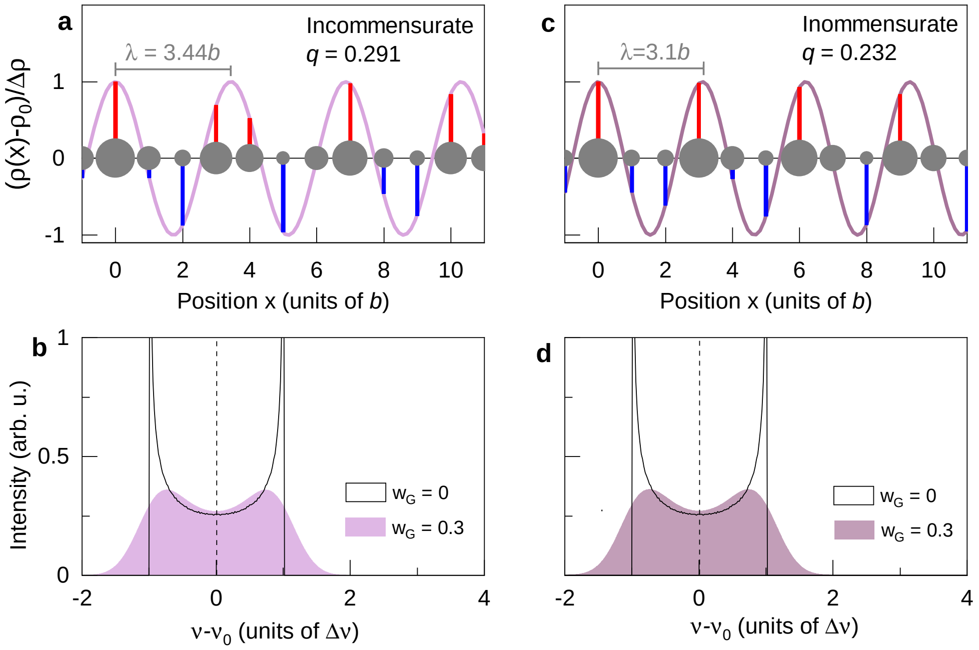

Supplementary Figure 1: For incommensurate modulations, the NMR line shape is independent of the value of the CDW wave vector q. This is illustrated here for $q = 0.291$ (panels a and b) and $q = 0.323$ (panels c and d). It is easier to visualise the large (actually infinite) number of inequivalent sites for $q = 0.291$ than for $q = 0.323$, when showing a 11 unit-cell long section (especially for the high charge-density - red - sites). To have the same visual effect with $q = 0.323$ would require to plot the modulation over about four times the length (40 unit cells).

Supplementary note 2: Phase dependence of commensurate CDWs

For incommensurate CDWs a global phase cannot be defined as the phase between the modulations maximum and the atomic position varies. On the other hand, an ideal commensurate modulation locks in with the lattice and the phase is fixed.

Supplementary Fig. 2 visualises how the choice of the phase affects the expected line shapes for unidirectional modulation with periods $\lambda = 3b$ and $\lambda = 4a$, respectively. It should be noted that depending on the phase of the modulation with period $3b$ the resulting histogram can be symmetric or asymmetric, however, for even periods like $4a$ the histogram is always symmetric, as can be easily seen from the polar plot of Supplementary Fig. 2m: For even periods to any atom sampling a positive charge amplitude there is another atom sampling a negative charge amplitude.

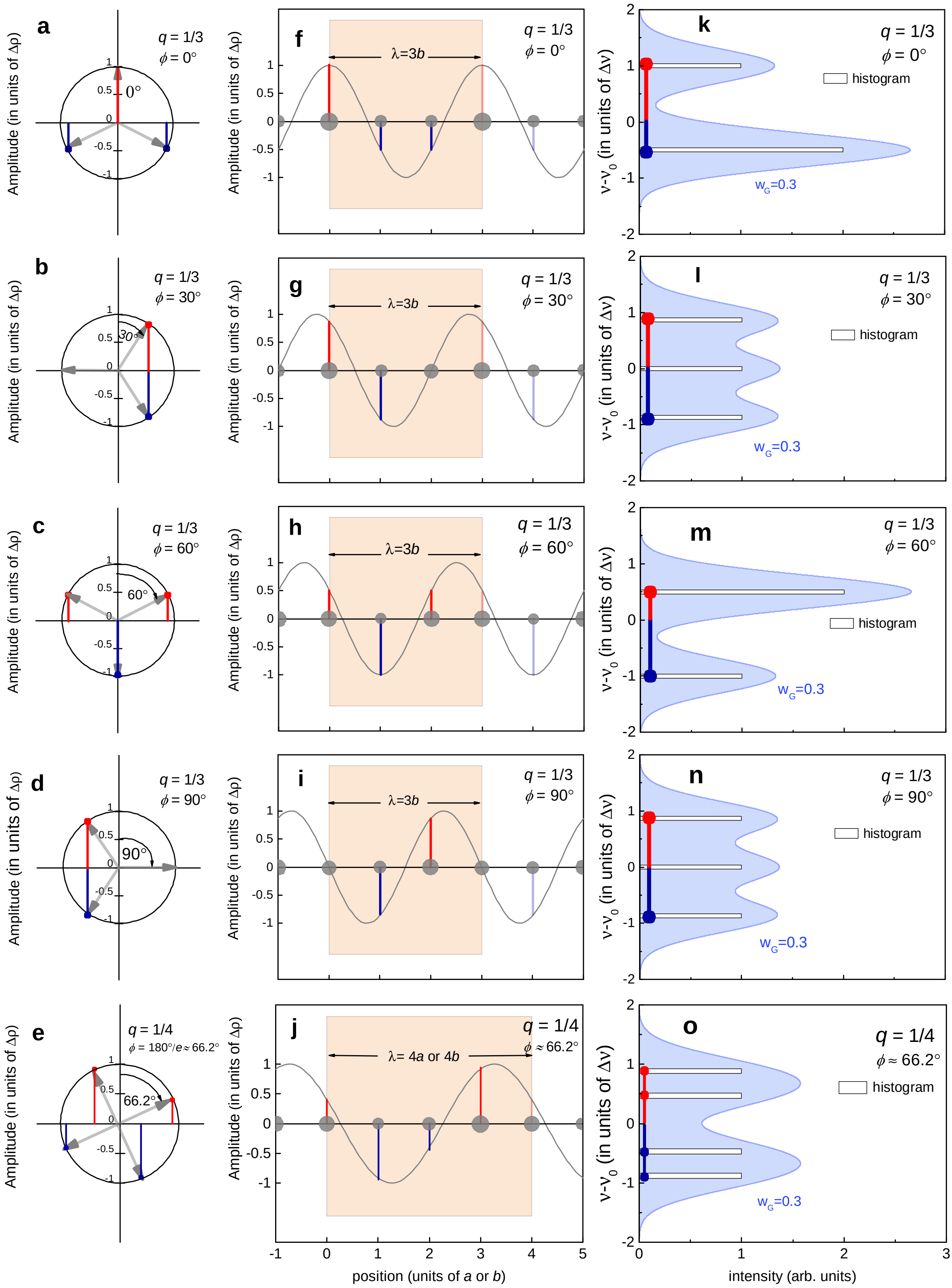

Supplementary Figure 2: Right-hand panels show the expected NMR spectra for commensurate modulations $\rho(x) = \rho_0 + \Delta\rho\cos(q \cdot x + \phi)$ with wave length $\lambda = 3b$ (**k-n**) and $\lambda = 4a$ (**o**). Spectra are based on histograms, convoluted with a Gaussian of width w_{G}= 0.3 (in units of $\Delta\nu$). The histograms are determined by sampling the modulation (vertical bars) at the atomic positions (grey circles) shown in central panels **f-j**. Atoms that experience a positive amplitude have a positive (red) shift $\nu - \nu_0$ in the NMR spectra. Negative shifts are blue. Atom sizes indicate the charge amplitude due to the CDW. Polar plots in **a-e** serve to visualise how the amplitude of the modulation at each atomic position changes with phase ϕ increasing from $\phi = 0°$ to 90° for $\lambda = 3b$ (**a-d**) and fixed at the irrational value $\phi = 180°/e$ for $\lambda = 4a$ (panel **e**), to show the most generic case. For $\phi = 45°$ there would be just two distinct sites. Atoms are represented by grey arrows of unit length. The arrows' projections on the vertical axis are equivalent to the cosinusoidal modulation's amplitude at the atomic position.

Supplementary Note 3: Bi-directional CDWs

A bi-directional modulation of the charge density takes the form:

$$\rho(x) = \rho_0 + \frac{\Delta\rho_a}{2}\cos(q_a \cdot x + \phi_a) + \frac{\Delta\rho_b}{2}\cos(q_b \cdot y + \phi_b)$$

No simple splitting is expected to result from an isotropic ($\Delta\rho_a = \Delta\rho_b$) bi-directional CDW as the modulation is sampled at more than two distinct positions. For commensurate modulations, histograms will have more peaks and these are more likely to overlap, thus leading to a broadened line shape, as shown in Supplementary Figs. 3a and 3b. For incommensurate modulations, the histogram has a single singularity centred at zero shift (Supplementary Fig. 3c), and thus no splitting either. Clearly, bi-directional modulations are inconsistent with the high-field NMR data.

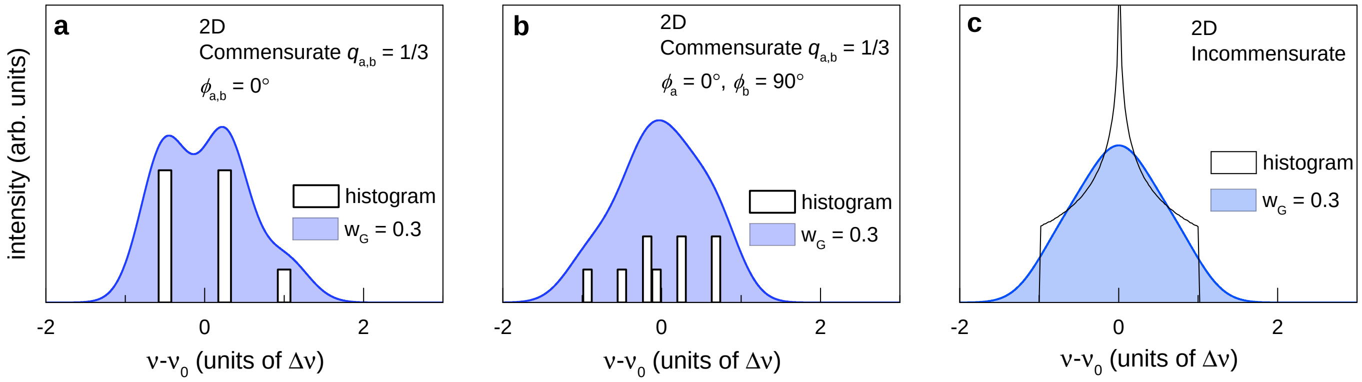

Supplementary Figure 3: Panels **a** and **b**: Histograms and simulated spectra of isotropic 2D commensurate CDW with $\lambda_a = 3a$ and $\lambda_b = 3b$ and equal ($\phi_{a,b} = 0°$) or differing ($\phi_a = 0°$, $\phi_b = 90°$) phases, respectively. Panel **c**: Histogram and broadened spectrum for an isotropic 2D incommensurate CDW.

Supplementary Note 4: Intra-unit-cell phase relationship from fitting of ^{17}O(3) NMR spectra

Full and empty oxygen chains lead to two distinct O(3) sites, O(3F) and O(3E), and two distinct Cu(2) sites, Cu(2E) and Cu(2F). However, the O(3E) and O(3F) NMR lines overlap at low temperature in the present experiments. In order to fit the O(3) lines, we use the following constraints. 1) Given the absence of line splitting for Cu(2E) sites [2], O(3E) satellites are also supposed not to split. 2) For O-II order at $p = 0.109$, O(3E) and O(3F) lines must have equal amplitudes and we assume they also have equal widths (as observed in the short-range CDW phase [4]). 3) The area ratio of the split O(3F) sites A and B is assumed to be 2:1, as found for O(2). 4) Asymmetric line shapes are assumed for all sites, which simplifies the fitting procedure. The O(3E) line might however not be asymmetric if it indeed does not split (the splitting and the asymmetry were found to be proportional for O(2) sites [5]). Reducing the asymmetry of O(3E) is not expected to significantly affect the accuracy of the fit but the splitting between A and B sites would somewhat increase. Fits with these constraints are shown in Supplementary Fig. 4a,b. An anti-phase (d symmetry) intra-unit-cell relationship between O(2) and O(3F) appears to be preferred by the fitting procedure (Fig. 4c), as can be seen from Supplementary Fig. 4a where O(3F) splits similarly to O(2), even though O(3F) sites are shifted by $b/2$ with respect to O(2). Nevertheless, a simulation with opposite O(3F) splitting (Supplementary Fig. 4b), corresponding to an in-phase relationship with O(2), is not much worse and thus cannot be fully excluded. These fits yield a quadrupole splitting of O(3F) lines that is about one third of that for O(2) lines, but as discussed above this value depends on the imposed constraints such as the line asymmetry. Therefore we can only conclude that the CDW amplitude at O(3) sites is of the same order of magnitude as at O(2) sites, though probably smaller by a factor 2-3.

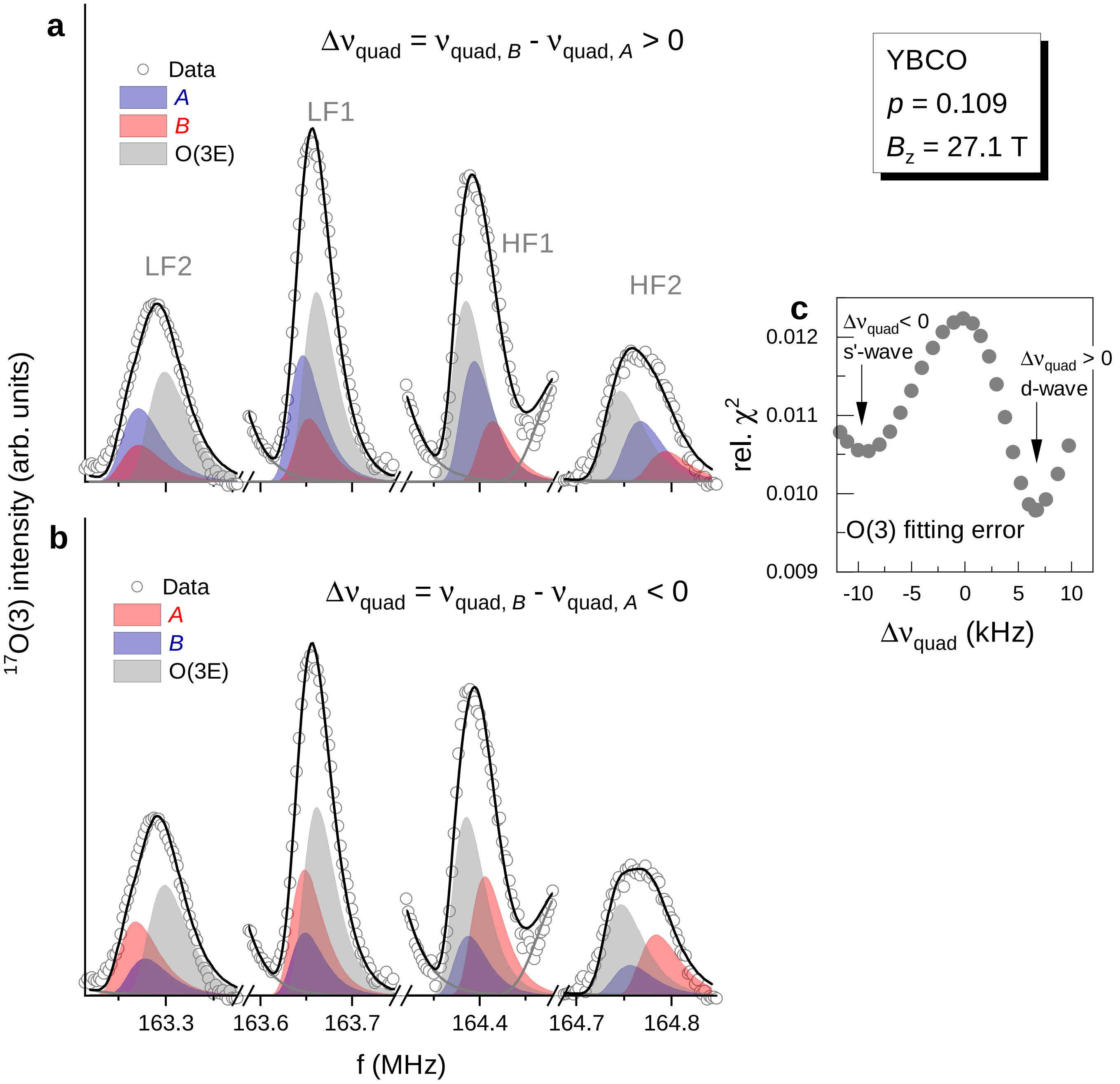

Supplementary Figure 4: **a**,**b** ^{17}O NMR spectrum of the O(3) satellites for the O-II ordered $p = 0.109$ sample at $B_z = 27.4$ T & 2.8 K. The sample was tilted by $\sim 16^\circ$ from the c axis towards a. Experimental data is fitted with three peaks: O(3E) and O(3F) A & B with equal widths and asymmetry. The area ratio is 3:2:1. The minority peak B has a larger (smaller) effective quadrupole frequency $\nu_{\rm quad}$ in **a** (**b**). Grey lines belong to O(2) satellites and need to be considered due to partial overlap with O(3). Panel **c** shows how the relative χ^2 error of the fit depends on the difference in $\nu_{\rm quad}$, $\Delta\nu_{\rm quad}$. Minima for $\Delta\nu_{\rm quad} < 0$ and $\Delta\nu_{\rm quad} > 0$ correspond to the fits shown in panels **b** and **a**, respectively.

Supplementary Note 5: Supporting data for commensurate period $\lambda = 3b$

Full fitted satellite ^{17}O-spectra of O(2) sites are shown in Supplementary Fig. 6 for all samples. The area ratio of the peaks A and B cannot be optimised in the *OriginPro* fitting-routine directly, as this poses a non-linear constraint. Fig. 5a shows that the fitting quality is optimal close to $A_A/A_B = 2 : 1$. As the peaks A and B correspond to different sampled charge environments, they have different quadrupole frequencies ν_{quad} (half the separation between the satellites LF1 and HF1). The variation of the quadrupole frequencies (with respect to $\nu_{\mathrm{quad,0}}$ outside of the high field phase, where the line shape is a single peak) is plotted against the field B_z in Supplementary Fig. 5b. Fig. 5c shows that the ratio of the quadrupole shifts $\Delta\nu_{\mathrm{quad,B}} / \Delta\nu_{\mathrm{quad,A}}$ field-independent. The average value is about -2 and lies within the experimental uncertainty to the expected ratio $-2 : 1$ for a commensurate period $\lambda = 3b$ and $\phi = 0°$. Other periods and phases would lead to both a different area ratio and a different quadrupole shift ratio.

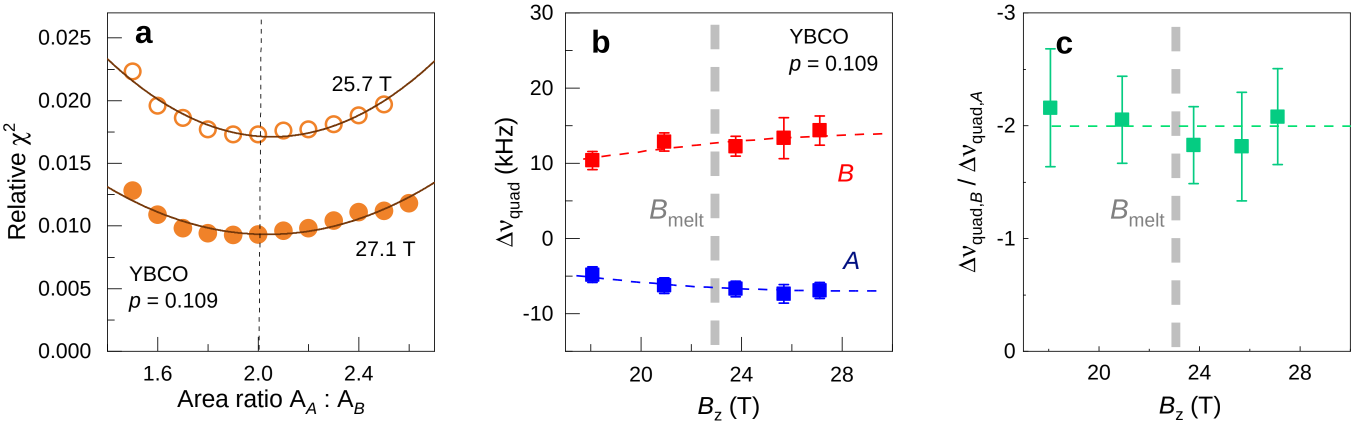

Supplementary Figure 5: Panel **a**: Relative χ^2 (squared residuals) of full ^{17}O NMR spectra at $B_z = 25.7$ T and 27.1 T for $p = 0.109$, respectively. The optimal area ratio is determined from the minimal relative χ^2 using quadratic fits (continuous lines). Panel **b**: Quadrupole shifts $\Delta\nu_{\mathrm{quad,A}}$ and $\Delta\nu_{\mathrm{quad,B}}$ vs. B_z for $p = 0.109$ as used to calculate the quadrupole shift ratio in panel **c** using Eq. 2). Panel **c** is identical to Main Fig. 4c. Thin dashes are guides to the eye while the thick vertical dashed line in panels **b** and **c** marks the vortex meting field at 2 K, $B_{\mathrm{melt}} \sim 23$ T. Error bars are defined as standard deviations from least squares fits.

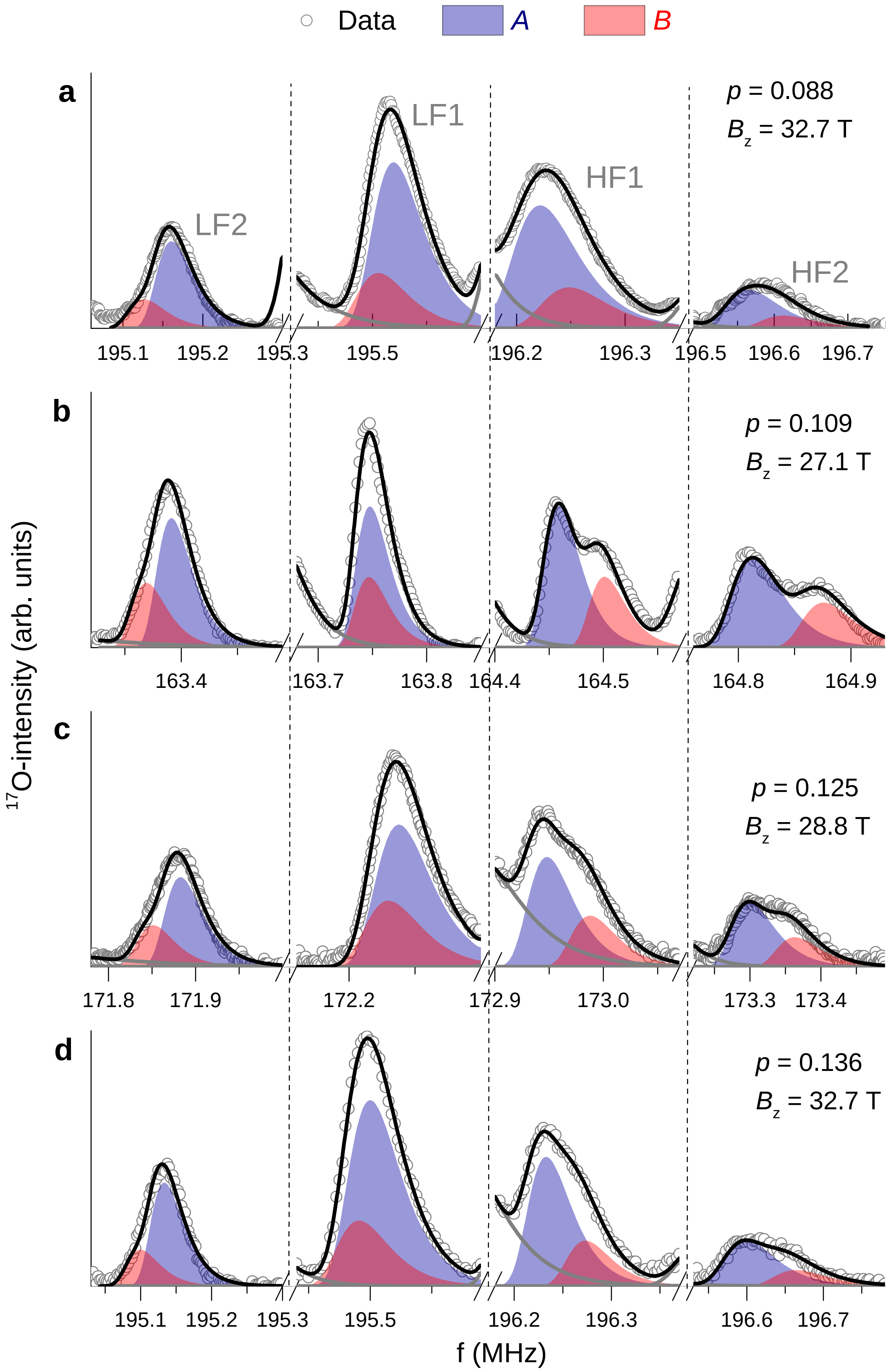

Supplementary Figure 6: ^{17}O NMR spectra of the O(2) satellites of four YBCO samples at very high fields & 2 K. Experimental data is fitted with two peaks (A & B) with equal widths while optimising the area ratio. Grey lines belong to O(3) satellites and need to be considered due to partial overlap with O(2). See Fig. 4b & d for fitting results. Dashed lines visualise axis breaks.

Supplementary Note 6: Quadratic dependence $\Delta\nu_{\text{tot}} \propto \Delta\rho^2$

In Fig. 1a of the main text, we have simulated the NMR spectrum for a unidirectional incommensurate modulation, assuming that the quadrupole frequency and the Knight shift are linear functions of the modulation of the charge density $\rho(x)$. In principle both $\Delta\nu_{\text{quad}}$ and ΔK can have a non-linear dependence on $\Delta\rho\cos(q\cdot x+\phi)$. We can include a quadratic term in the spirit of a Taylor expansion up to second order and write

$$\nu_{\text{tot}}(x) = \nu_0 + \Delta\nu_{\text{tot}}(x) = \nu_0 + \gamma B\Delta K(x) + n\cdot\Delta\nu_{\text{quad}}(x)$$
$$= \nu_0 + (a_K^{(1)} + a_Q^{(1)})\Delta\rho(x) + \frac{(a_K^{(2)} + a_Q^{(2)})}{2}\Delta\rho(x)^2$$

where the linear and quadratic coefficients $a^{(1)}$ and $a^{(2)}$ result from linear and quadratic quadrupole shifts and Knight shifts which combine differently depending on the particular quadrupole satellite (n) as $a^{(1)} = a_K^{(1)} + n\cdot a_Q^{(1)}$ for the linear component and correspondingly as $a^{(2)} = a_K^{(2)} + n\cdot a_Q^{(2)}$ for the quadratic component. We find that we can describe the splitting on the HF2 and HF1 satellites with a unidirectional incommensurate wave vector $q = 0.323$ and $a^{(1)} \approx a^{(2)}$ in units of $a^{(1)}$ of the HF2 satellite since the large quadratic component leads to a histogram with singularities of different amplitudes. Although these simulations work rather well and could be improved by adding cubic and higher orders, a strongly non-linear dependence on the charge density and thus the doping p is neither expected for the Knight shift, nor the quadrupole frequency, as on average, linear increase of ν_{quad} with hole doping seems to be equally correct for electron doped [6] (where ν_{quad} decreases with electron doping) and hole doped cuprates, both for in-plane Cu(2) and O(2) & O(3) sites [7–9]. Supplementary Fig. 7 shows that the quadratic contribution to the Knight shift, $a_K^{(2)}$, surpasses the linear contribution. However, experimentally the Knight shift increases only moderately with doping and tends to saturate near optimal doping, as can be seen from low-temperature Knight shift data of Zhou *et al.* [10]. As a commensurate modulation with $\lambda = 3b$ and $\phi = 0°$ fits the full spectrum consistently without the need of a non-linear dependence of Knight shifts and quadrupole shifts, we do not discuss incommensurate modulations any further.

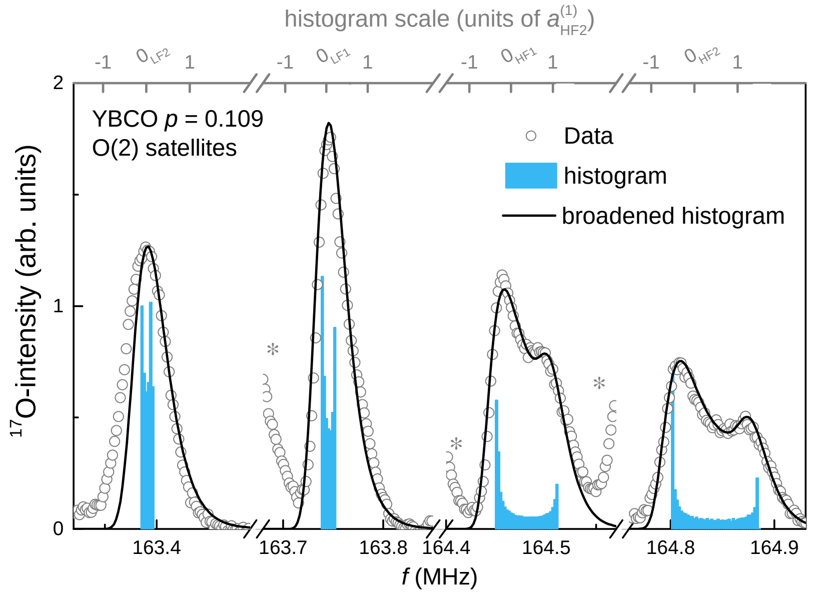

Supplementary Figure 7: Open circles mark NMR spectra of the O(2) satellites of the $p = 0.109$ YBCO sample at $B_z = 27.1$ T & 2 K. The satellites are modelled by broadening a histogram (blue bars) for a unidirectional incommensurate modulation by an asymmetric (extreme value distribution) line shape (black line). For HF2 the modelling is best if the total shift $\Delta\nu_{\text{tot}}(x)$ has nearly equal linear and quadratic contributions in the charge density ($a^{(2)} = 0.9a^{(1)}$). Modelling of HF2 and HF1 independently allows to extract $a_K^{(1)} = 0.44$, $a_K^{(2)} = 0.54$ and $a_Q^{(1)} = 0.56$, $a_Q^{(2)} = 0.36$. The low-frequency side of the LF2 satellite is not modelled properly for parameters determined from HF2 and HF1. Asterisks mark intensity from partially overlapping O(3) satellites.

Supplementary References

[1] Wu, T. *et al.* ^{63}Cu-NMR study of oxygen disorder in ortho-II $YBa_2Cu_3O_y$, Phys. Rev. B **93**, 134518 (2016).
[2] Wu, T. *et al.* Magnetic-field-induced charge-stripe order in the high-temperature superconductor $YBa_2Cu_3O_y$, Nature **477**, 191194 (2011).
[3] Blackburn, E. *et al.* X-Ray Diffraction Observations of a Charge-Density-Wave Order in Superconducting Ortho-II $YBa_2Cu_3O_{6.54}$ Single Crystals in Zero Magnetic Field, Phys. Rev. Lett. **110**, 137004 (2013).
[4] Wu, T. *et al.* Incipient charge order observed by NMR in the normal state of $YBa_2Cu_3O_y$, Nat. Commun. **6**, 6438 (2015).
[5] Zhou, R. *et al.* Quasiparticle Scattering off Defects and Possible Bound States in Charge-Ordered $YBa_2Cu_3O_y$, Phys. Rev. Lett. **118**, 017001 (2017).
[6] M. Jurkutat, PhD thesis, NMR of Electron-Doped High-Temperature Superconductor $Pr_{2-x}Ce_xCuO_4$, Universität Leipzig (2014).
[7] G.-q. Zheng, Y. Kitaoka, K. Ishida, K. Asayama, Local Hole Distribution in the CuO_2 Plane of High-T_c Cu-Oxides Studied by Cu and Oxygen NQR/NMR, J. Phys. Soc. Jpn. **64**, 2524 (1995).
[8] M. Jurkutat, J. Kohlrautz, S. Reichardt, A. Erb, G. V. M. Williams, and J. Haase, NMR of Cuprate Superconductors: Recent Developments, High-Tc Copper Oxide Superconductors and Related Novel Materials, Springer (2017).
[9] J. Haase, O. P. Sushkov, P. Horsch, G. V. M. Williams, Planar Cu and O hole densities in high-T_c cuprates determined with NMR, Phys. Rev. B **69**, 094504 (2004).
[10] Zhou, R. *et al.* Spin susceptibility of charge-ordered $YBa_2Cu_3O_y$ across the upper critical field, Proc. Natl. Acad. Sci. USA **114**, 13148-13153 (2017).
